# Identification of clinical phenotypes in knee osteoarthritis: a systematic review of the literature

**DOI:** 10.1186/s12891-016-1286-2

**Published:** 2016-10-12

**Authors:** A. Dell’Isola, R. Allan, S. L. Smith, S. S. P. Marreiros, M. Steultjens

**Affiliations:** Institute of Applied Health Research/School of Health and Life Sciences, Glasgow Caledonian University, Glasgow, G4 0BA Scotland, UK

**Keywords:** Knee, Osteoarthritis, Phenotype, Sub-group, Clinical

## Abstract

**Background:**

Knee Osteoarthritis (KOA) is a heterogeneous pathology characterized by a complex and multifactorial nature. It has been hypothesised that these differences are due to the existence of underlying phenotypes representing different mechanisms of the disease.

**Methods:**

The aim of this study is to identify the current evidence for the existence of groups of variables which point towards the existence of distinct clinical phenotypes in the KOA population. A systematic literature search in PubMed was conducted. Only original articles were selected if they aimed to identify phenotypes of patients aged 18 years or older with KOA. The methodological quality of the studies was independently assessed by two reviewers and qualitative synthesis of the evidence was performed. Strong evidence for existence of specific phenotypes was considered present if the phenotype was supported by at least two high-quality studies.

**Results:**

A total of 24 studies were included. Through qualitative synthesis of evidence, six main sets of variables proposing the existence of six phenotypes were identified: 1) chronic pain in which central mechanisms (e.g. central sensitisation) are prominent; 2) inflammatory (high levels of inflammatory biomarkers); 3) metabolic syndrome (high prevalence of obesity, diabetes and other metabolic disturbances); 4) Bone and cartilage metabolism (alteration in local tissue metabolism); 5) mechanical overload characterised primarily by varus malalignment and medial compartment disease; and 6) minimal joint disease characterised as minor clinical symptoms with slow progression over time.

**Conclusions:**

This study identified six distinct groups of variables which should be explored in attempts to better define clinical phenotypes in the KOA population.

**Electronic supplementary material:**

The online version of this article (doi:10.1186/s12891-016-1286-2) contains supplementary material, which is available to authorized users.

## Background

Osteoarthritis is the most common form of arthritis; it constitutes a leading cause of disability in the adult population [1] with the knee the most affected joint. Knee Osteoarthritis (KOA) is a heterogeneous pathology characterized by a complex and multifactorial nature [2]. This multifactorial aetiology contributes to the broad variation in symptoms presentation and treatment response that characterize the KOA subjects and constitutes a challenge for the identification of personalized and effective interventions. Therefore, in order to optimize treatment effect in KOA, the intervention should address this variability and should be tailored to specific subgroups or phenotypes as highlighted in the NICE guidelines on KOA [3–6]. A phenotype in KOA can be defined as a collection of observable traits (i.e. aetiologic factors, risk factors) that can identify and characterize a subgroup in a defined population. The presence of distinct phenotypes within the KOA patient population would suggest distinct underlying causes and mechanisms, which could be highly relevant for understanding and treating the disease [7, 8].

Previous attempts to identify distinctive KOA phenotypes used different perspectives. Some researchers used disease progression to determine KOA phenotypes, while others looked at pain perception or the degeneration pattern of the cartilage [9–15]. Potentially, hundreds of phenotypes may be identified depending on the definition of phenotypes and on the variables selected. Each approach can be considered equally valid depending on the scope. Only studies focusing on the identification of clinical subgroups characterized by different disease mechanisms can be considered useful to improve treatment allocation and clinical management of the disease. If, as hypothesized, treatments Are highly effective only in one sub-type; the therapeutic effect of the intervention will be lost if tested in KOA population as a whole [4]. Therefore, the identification of risk and aetiologic factors that can identify specific clinical subtypes of KOA is an important starting point for the implementation of phenotyping research in clinical practice and may be critical for the improvement of treatment allocation and for the development of new treatment strategies.

The aim of this review is therefore to synthesize the current evidence for the existence of distinct sets of variables that may suggest the existence of clinical KOA phenotypes characterized by the presence of different risk and aetiologic factors.

## Methods

### Information sources

A systematic literature search was conducted in PubMed (Medline) for the period from 01/01/1984 to 29/04/2016. An additional manual search was completed by ADI from the references of the selected papers.

The research strategy was built up using the following key words: osteoarthritis, knee, phenotyp*, subgroup, cluster, “factor analysis”. These terms were combined in the following way: osteoarthritis AND knee AND (phenotyp* OR subgroup OR cluster OR “factor analysis”) (for further details see Additional file 1-A).

### Inclusion criteria

Articles were included if: (1) the population involved (a subgroup of) patients over 18 years of age; (2) the population consisted of patients diagnosed with KOA; (3) the aim was to identify clinical phenotypes of patients with KOA; (4) the methodology and analysis were designed to identify phenotypes (e.g. cluster analysis using clinical variables) ; (5) the article was an original research report. Previous systematic reviews were excluded. In addition to the second criterion, studies that included patients with hip or hand OA other than KOA were included if: (1) they used biomarkers or other measures that are not joint specific, (2) the KOA subgroup represented more than 60 % of the sample.

### Data selection process

Article selection was made independently by two reviewers (ADI and MS) based on title and abstract according to the inclusion criteria. The final selection was made by the same two independent reviewers based on the full text. Disagreements between the two reviewers were resolved by the intervention of a third reviewer (SS); this procedure was adopted for both selection steps.

### Assessment of methodological quality (risk of bias)

The methodological quality of the papers was assessed using an adaption of the standardized Hayden score [16] (Table 1) to identify the risk of bias affecting the validity of findings. All papers were reviewed by ADl and MS, with additional proportional reviews performed by RA, SM, SS using block allocation with each reviewing 2/3 of the final papers.

**Table 1:** Adaptation of the Hayden score for the evaluation of the risks of bias

Areas of potential bias	Explanation and Adaptation
(1) Participation	Source population and characteristic of the sample
(2) Study attrition	Loss to follow up
(3) Measurement of prognostic factors^a^	A clear definition or description of the prognostic factor measured is provided and adequately reported. Adaptation: We considered as prognostic factor the variable chosen in the study to classify the patients and define the phenotypes
(4) Outcome measurement^a^	A clear definition of the outcome of interest is provided and the outcome methods are valid and reliable. Adaptation: we considered the variable used to define the difference between subgroups as outcome measures
(5) Confounding factors	Are confounders present in the study; confounding factors are accounted for in the study design
(6) Analysis	Data analysis and data presentation

^a^: areas of potential bias adapted to match the design of the studies included

The risk of bias for each area was rated as low, moderate or high [16]. Studies that had a high risk of bias in at least one of the area assessed were considered to have an overall high risk of bias and regarded as sources of low quality evidence used only to support the findings of other (i.e. high quality) studies. Studies with low to moderate risk of bias and appropriate design were considered sources of strong evidence.

### Data-extraction analysis

The data from each study were extracted by two reviewers (ADI, MS) and included number of patients, clustering method and subgroups identified. Additionally, the prevalence of each phenotype was extracted where possible.

### Identification of phenotypes

In this systematic review we adopted a tailored data analysis process in order to deal with the broad variation in the methodologies of the studies included. This process shares some similarities with the directed content analysis method [17]. Key variables for each phenotype reported in the included studies were extracted. Using the theory and previous evidence, we assigned each key variable to a category (e.g. biomechanical, inflammatory, metabolic) indicating the underlying disease mechanism represented by that specific variable.. Variables (e.g. radiographic features, pain sensitization) were considered to suggest similar disease mechanisms and classified in the same category if: (1) It was specifically stated by the author of the paper (e.g. two subgroups extracted from two different studies were reported by the respective authors as representing the same phenotype); (2) the association of the reported characteristic to a specific pathophysiologic mechanism had been reported in previous studies investigating disease mechanisms and risk factors (e.g. malalignment consistent with compartment degeneration has previously been reported as a biomechanical mechanism responsible for KOA development). Each phenotype was then classified in the category indicated by the variable that characterized it. A phenotype was considered supported by evidence when at least two studies with low risk of bias identified a phenotype under the same category. If a phenotype was reported in only a single study this was not considered sufficient evidence to include this phenotype in the final list of phenotypes identified in this review.

## Results

### Description of the included studies

**Fig. 1:**
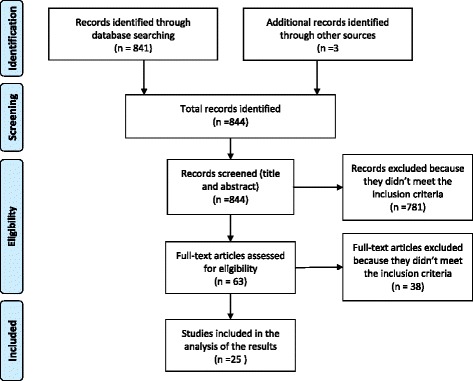
Flow chart of the study selection process for eligible studies in the systematic review

The initial literature search identified 841 articles. Three additional papers were identified through a manual search of the references. After screening for title and abstract, 781 papers were excluded. The full texts for the remaining 63 articles were assessed for inclusion. From this list, 25 articles matched the inclusion criteria and were included in the systematic review (Fig. 1) [7–9, 18–39]. For an overview of the studies included see Table 2. (For further details see Additional file 1)

**Table 2:** Description of the papers

Author	Type of research	Type of study	Analysis	Participants	Control	Subgoups					
						Chronic pain	Inflammatory	Metabolic syndrome	Bone and cartilage metabolism	Mechanical overload	Minimal joint disease
Attur 2011 [18]	Genetic/gene expression	Cohort (prosp)	complete-linkage hierarchical clustering	1: 41^a^ 2: 36^a^ 3: 86^a^	1: 25^a^ 2: 0^a^ 3: 12^a^	-	1: 16/41 = 39 %. 2: 8/36 = 22 %, 3: 33/86 = 38 %	-	-	-	-
Bae 2010 [19]	Imaging (photography)	Cross sectional	K-means cluster analysis	127	-	-	-	-	-	20 %^b^	-
Berry 2010a [20]	Biomarker	Cohort (prosp)	Mann–Whitney u, χ^2^, Multiple regression analysis	117	-	-	-	-	Prevalence not reported	-	-
Berry 2010b [21]	Biomarker	Cohort (prosp)	Mann–Whitney u, Multiple regression and logistic regression analysis	117	-	-	-	-	-	-	Prevalence not reported
Blumnenfeld 2013 [22]	Biomarker	Cohort (prosp)	Binary logistic regression analysis	Different in different analysis	Different in different analysis	-	-	-	Prevalence not reported	-	-
Cruz-Almeida 2013 [23]	Lab experimental (non-biomech)	Cross-sectional	Hierarchical cluster analysys	194	-	32/194 = 16 %	-	-	-	-	-
Doss 2007 [24]	Biomarker	Cross-sectional	Mann–Whitney	49	-	-	8/49 = 16 %	-	-	-	-
Egsgaard 2015 [25]	Biomarker	Case control	Principal component analysis/Hierarchical cluster analysis	216	64	41/212 = 19 %	-	-	-	-	-
Fernández-Tajes 2014 [26]	Genetics	Case control	Cluster analysys (unsupervised)	23	18	-	7/23 = 30 %	-	-	-	-
Holla 2013 [27]	Epidemiology	Cohort (prosp)	Latent class growth analysis	697	-	-	-	-	-	-	330/697 = 47 %
Jenkins 2015 [28]	Epidemiology	Secondary data analysis	Hierarchical and k -means cluster analysis	75	-	-	-	-	-	-	Prevalence not reported
Kerkhof 2008 [29]	Genetics	Cross sectional	χ^2^, OR, ANCOVA, meta-analysis of existing cohorts	4993	-	-	-	-	-	-	-
Kinds 2013 [9]	Imaging	Cohort (prosp)	Hierarchical cluster analysys	336	-	-	-	-	-	-	108/417 = 26 %
King 2013 [30]	Lab experimental (non-biomech)	Case control	ANCOVA	209	107	Subgroups splitted using mean value of womac (percentage not reliable)	-	-	-	-	-
Knoop 2011 [7]	Epidemiology	Secondary data analysis	K-means luster analysis	842	-	83/841 = 10 % (only depression)	-	168/841 = 22 % (only obese)	-	189/841 = 22 %	140/841 = 17 %
Murphy 2011 [31]	Epidemiology	Cross-sectional	Hierarchical cluster analysis	129	-	45/125 = 36 %	-	-	-	-	-
Otterness 2000 [32]	Biomarker	Case control	Principal component analysis	39	21	-	Prevalence not reported	-	Prevalence not reported	-	-
Pereira 2013 [33]	Epidemiology	Cross-sectional	T-test, OR, logistic regression	663	-	Prevalence not reported	-	-	-	-	-
Roemer 2012 [34]	Imaging	Cross sectional	OR	1248	-	-	-	-	1248 subjects/0,2 % hypertrophic-1.3 % atrophic	-	-
Sowers 2002 [35]	Biomarker	Cohort	ANOVA, χ^2^	1025	-	-	-	11 %^b^	-	-	-
Van der Esch 2015 [36]	Epidemiology	Secondary data anlysis	K-means cluster analysis	551	-	86/551 = 15.6 % (only depression)	-	81/551 = 15 % (only obese)	-	114/551 = 20.6 %	154/551 = 28 %
Van Spil 2012 [37]	Biomarker	Cohort (prosp)	Principal component analysis, multiple linear regression (interaction terms)	1002	-	-	Prevalence not reported	-	Prevalence not reported	-	-
Waarsing 2015 [8]	Epidemiology	Secondary data analysis	Latent class cluster analysis	518	-	-	-	27 % (group with hypertension and higher BMI)	-	15 % (lateral degeneration) 12 %(previous injuries)	47 %^b^
Iijima 2015 [38]	Epidemiology	Cross sectional	Multiple Logistic regression Analysis	266	-	-	-	-	-	26/266 = 9.7 % (static + dinamic malalignment)	-
Kittelson 2015 [40]	Epidemiology	Secondary data analysis	Latent class analysis	3494	-	337/3494 = 9.6 %	-	-	-	-	-

^a^: this study is composed of 3 cohorts, the results obtained in the first cohort were replicated in the other two to validate the results

^b^: Only percentage reported

.

### Quality assessment

The quality assessment resulted in 21 papers with low or moderate risk of bias (considered to be high quality studies) and four papers with a high risk of bias for at least one of the six areas reviewed, which were considered to be low quality studies (Table 3).

**Table 3:** Risk of bias assessment adapted from Hayden et al

	Risk of Bias						
Author	Participation	Attrition	Prognostic Factors	Outcome	Confounding	Analysis	Total Score
Attur 2011 [18]	Low	Low	Low	Low	Moderate	Low	Low
Bae 2010 [19]	Moderate	N/A	Low	Low	Moderate	Low	Low
Berry 2010a [20]	Moderate	Moderate	Low	Low	Moderate	Moderate	Low
Berry 2010b [21]	Low	Low	Low	Low	Moderate	Low	Low
Blumnenfeld 2013 [22]	Low	Moderate	Moderate	Moderate	Moderate	Moderate	Low
Cruz-Almeida 2013 [23]	Moderate	N/A	Low	Low	Moderate	Low	Low
Doss 2007 [24]	Moderate	N/A	Low	Moderate	Moderate	Low	Low
Egsgaard 2015 [25]	Moderate	N/A	Low	Low	High	Low	High
Fernández-Tajes 2014 [26]	Moderate	N/A	Low	Moderate	Moderate	Moderate	Low
Holla 2013 [27]	Moderate	Low	Low	Low	Low	Low	Low
Jenkins 2015 [28]	High	N/A	Moderate	Moderate	High	Moderate	High
Kerkhof 2008 [29]	Low	Low	Low	Low	Moderate	Low	Low
Kinds 2013 [9]	Moderate	Low	Low	Low	Moderate	Moderate	Low
King 2013 [30]	High	N/A	High	Low	High	Low	High
Knoop 2011 [7]	Low	N/A	Low	Low	Low	Moderate	Low
Murphy 2011 [31]	Moderate	N/A	Low	Moderate	Moderate	Low	Low
Otterness 2000 [32]	Moderate	N/A	Low	Moderate	Moderate	Moderate	Low
Pereira 2013 [33]	Low	N/A	Moderate	Low	Moderate	Low	Low
Roemer 2012 [34]	Low	N/A	Low	Low	Moderate	Moderate	Low
Sowers 2002 [35]	Moderate	Low	Low	Low	Moderate	Moderate	Low
Van der Esch 2015 [36]	Low	N/A	Low	Low	Moderate	Low	Low
Van spil 2012 [37]	Moderate	N/A	Low	Low	Moderate	Low	Low
Waarsing 2015 [8]	Low	N/A	Low	Low	Low	Low	Low
Iijima 2015 [38]	Moderate	N/A	Low	Low	High	Low	High
Kittelson 2015 [40]	Low	N/A	Low	Low	Low	Low	Low

*N/A* not applicable, the specific area of assessment was not applicable to the study

### Phenotypes

A total of 79 phenotypes were reported in the included studies. Of those, 42 phenotypes were reported in a single study only and therefore not taken forward into the qualitative evidence synthesis. The remaining 37 subgroups were matched and combined into six main groups of variables that suggest the existence of different mechanisms in the KOA population: chronic pain; inflammatory mechanisms; metabolic mechanisms of bone and cartilage local to the joint, metabolic syndrome; mechanical overload; and minimal joint disease (Additional file 1: Table S1 and S2). These mechanisms may be responsible for the disease in specific subgroup or phenotypes. The six main sets of variables that emerged from the literature are indicative of different disease aetiology with the exception of the minimal joint disease phenotype that classifies the subjects based on the disease progression. Only one paper reported negative results, founding no evidence for the existence of distinct phenotypes within the KOA patient population [29]. An overview of the variables extracted from each paper is provided in Table 2 and in the Additional file 1.

### Chronic pain

Six cross sectional studies with low risk of bias, [7, 23, 31, 33, 36, 40] indicating the central nervous system and alterations in pain neurophysiology as key factors in the disease pathophysiology, were considered to support a chronic pain phenotype. Two additional studies with high risk of bias demonstrated similar findings. A chronic pain phenotype was defined using variables associated with central sensitisation [23, 40] (e.g. quantitative sensory testing (QST) [23]); pain and psychological profiling [31, 33]. The included studies demonstrated a high prevalence of lower pain pressure threshold and enhanced mechanical pain responses to temporal summation in several sites, suggesting full manifestation of peripheral spreading and central sensitization in a particular subgroup of KOA subjects. Moreover, the presence of psychological distress; poor coping style; sleep disturbance; fatigue; widespread pain and illness burden signify the existence of complex mechanisms that involve the entire body rather than the knee as the “target” of the disease [23, 25, 31, 40]. These features have a prevalence of 16 % to 19 % in the KOA samples used in the aforementioned studies.

### Inflammatory KOA

Two cohort studies and three cross-sectional studies with low risk of bias identified specific subgroups of patients suggesting the existence of an inflammatory KOA phenotype, the prevalence of which varies in the different samples from 16 % to 30 % [18, 24, 26, 32, 37]. Attur et al. found a subgroup of KOA patients in which a gene overexpression of inflammatory cytokines is present (Interleukin-1B [IL-1β], Interleukin-8 [IL-8], cycloxygenase 2[COX-2], GRO 2, macrophage inflammatory protein-1α [MIP-1α] and -1β [MIP-1β]) [18]. These patients had a higher level of pain at the baseline and experienced faster radiographic progression compared to the group with cytokine underexpression. Furthermore a higher cytokine IL-6 concentration in the synovial fluid has been found in a subgroup of people undergoing a total knee (and hip) replacement [24]. The same cytokine has also been found to be associated with other inflammation markers (C-reactive protein [CR-P], tumor necrosis receptor type I [TNFI] and tumor necrosis receptor type II [TNFII], eosinophilic cationic protein [ECP]) in an inflammatory phenotype identified through a serum analysis [32]. Other inflammatory biomarkers (Serum III procollagen peptide [sPIIINP], serum hyaluronic acid [sHA], sCOMP) characterise a subgroup found by van Spil et al. with a higher synovial activity [37].

### Metabolic syndrome

A metabolic phenotype characterized by variables suggesting that a systemic metabolic syndrome contributes significantly to the disease was supported by the literature Two longitudinal studies and two cross-sectional studies, all with low risk of bias (Tables 2, 3 and 4) [7, 35–37], suggest the existence of specific subgroups of patients characterized by a higher prevalence of metabolic factors (obesity, diabetes, hypertension and dyslipidemia) and a specific biomarker profile (plasma leptin [pLeptin], High-Sensitivity C-RP, erythrocyte sedimentation rate [ESR]) [7, 35–37]. These findings suggest the existence of a metabolic syndrome phenotype.

**Table 4:** Appraisal of the evidence

	Phenotypes					
Author/year	Chronic pain	Inflammatory	Metabolic syndrome	Metabolic bone/cartilage	Mechanical overload	Minimal joint disease
Attur 2011 [18]		++				
Bae 2010 [19]					++	
Berry 2010a [20]				++		
Berry 2010b [21]						++
Blumnenfeld 2013 [22]				++		
Cruz-Almeida 2013 [23]	++					
Doss 2007 [24]		++				
Egsgaard 2015 [25]	+					
Fernández-Tajes 2014 [26]		++				
Holla 2013 [27]						++
Jenkins 2015 [28]						+
Kerkhof 2008 [29]						
Kinds 2013 [9]						++
King 2013 [30]	+					
Knoop 2011 [7]	++		++		++	++
Murphy 2011 [31]	++					
Otterness 2000 [32]		++		++		
Pereira 2013 [33]	++					
Roemer 2012 [34]				++		
Sowers 2002 [35]			++			
Van der Esch 2015 [36]	++		++		++	++
Van Spil 2012 [37]		++	++			
Waarsing 2015 [8]				++	++	++
Iijima 2015 [38]					+	
Kittelson 2015 [40]	++					
Total number of studies	6 (2)	5	4	5	4 (1)	6 (1)

+ high risk of bias, ++ low risk of bias

Total Number of Studies: low risk of bias (high risk of bias)

### Bone and cartilage metabolisms

Five studies (three longitudinal studies; two cross-sectional studies) reported subgroups of participants with alterations in bone and cartilage metabolism within the knee joint [20, 22, 32, 34, 37]. Berry et al. found an association between CTX-I; NTX-I and reduced cartilage lost in two subgroups, one characterized by high levels of osteocalcin and the other by high levels of PINP [20]. Van Spil et al. and Blumenfeld et al. found a cluster of biomarkers associated with bone and cartilage metabolism (uCTX-I, uCTX-II, uNTX-I, sPINP, sOC, sCOMP) [22, 37]. Additionally, Otterness et al. identified three metabolic subgroups using bone markers (bone sialoprotein, hydroxylysyl pyridinoline, lysyl pyridinoline [BSP, HP, LP], putative markers of cartilage anabolism (carboxypropeptide of type II [CPII], HA, epitope 846) and catabolism (keratan sulfate [KS], COMP) [32]. Roemer et al. used MRI to identify two rare phenotypes characterised by hypertrophic and atrophic reactions of the bone with a prevalence of 0.2 %-1.3 % respectively [34]. Evidence from these papers also suggests this may constitute more than a single phenotype, including some rare variations.

### Mechanical overload

Four cross sectional studies with low risk of bias and one with high risk of bias reported biomechanical factors as main mechanisms of the disease in specific KOA populations [7, 8, 19, 36]. From the data extracted, these mechanisms seem responsible for the disease in 12 %-22 % of the KOA population. Waarsing et al. identified two subgroups in which biomechanical stressors appear to be responsible for the disease [8]. One subgroup was characterized by degeneration of the lateral compartment, valgus alignment, and lower BMI while a high prevalence of previous injuries (55 %), a severe degeneration of cartilage in the medial compartment and varus malalignment represent the main features in the other group. Knoop et al. and van der Esch et al. found, using cluster analysis, a phenotype with strong muscle strength, severe degeneration and low BMI [7, 36], while Bae et al. identified two subgroups of KOA patients with full thickness cartilage lesions in the medial and patella-femoral compartments [19]. Overall, this phenotype appears to be characterized by excessive mechanical forces acting on specific areas within the joint, causing KOA.

### Minimal joint disease

Six studies with low risk of bias, of which three had a longitudinal design and three a cross sectional design, suggest the existence of a subgroup of KOA patients whereby the disease is characterised by low degeneration, mild clinical symptoms and slow progression over time (2–10 years) [7–9, 21, 27, 36]. One further study with high risk of bias reported similar findings. Among these seven studies, a combination of magnetic resonance imaging (MRI) and biomarkers (serum cartilage oligomeric protein [sCOMP] , N-terminal propeptide of collagen IIA [PIIANP]) [21]; x ray and clinical data [9, 27], and cluster analysis (upper leg muscle strength, body mass index (BMI), severity of radiographic OA, depressive mood, radiographic scores of OA features, regional quantitative MRI measures of cartilage and bone, and self-reported knee symptoms) [7, 36] were used to determine the severity of the disease. Subjects were classified in this phenotype according to the actual status of the disease and the long term outcome (2–10 years). This phenotype represents the only subgroup defined without regard to the disease aetiology. Five studies reported or allowed the calculation of the prevalence in the KOA population of these features that varied between 17 % and 47 %.

## Discussion

The aim of the present study was to synthesize the current evidence for the existence of clinical phenotypes in the KOA population. Six main groups of variables which suggest the existence of different underlying disease mechanisms in the KOA population were identified after a qualitative data analysis. These sets of variables should be further explored in order to confirm and better define the KOA phenotypes emerging from the literature.

In the chronic pain phenotype, high prevalence of widespread pain and psychological disturbs suggests that central sensitization plays a fundamental role in the disease process. Severe pain is often reported in association with low or moderate degeneration of the local joint structures. In these subjects, the joint disease alone is not sufficient to explain the complex symptomatology, thus it is likely that these subjects belong to a specific KOA phenotype rather than to a stage of the disease [7, 36]. Due to the reversibility of central sensitization combined with the lack of longitudinal studies, it is not yet clear if membership of this subgroup is stable over time. Despite this uncertainty, when patients present symptoms consistent with a chronic pain phenotype, they may need and respond to treatments that differ from those targeted towards joint pain [4]. Cognitive-behavioural therapy and pain education can be worthwhile in this phenotype and may optimize the results of other traditional intervention such as exercise therapy and joint replacement [23].

In recent years, a growing body of evidence supports the involvement of local inflammatory mediators in the disease pathogenesis [41]. Signs of inflammation have been found in a large part of the KOA population. In many cases these signs seem only to characterize specific phases of the disease [42]. From this literature review emerged evidence that a subgroup of the KOA subjects presents specific inflammatory mechanisms as determinant of the disease. Attur et al. identified a group of KOA subjects with a gene overexpression of inflammatory cytokines in a study with longitudinal design [18]. This finding suggests that KOA subgroups characterized by specific inflammation mechanisms may exist regardless of disease stage, as found in other studies [43, 44]. Treatments targeting the inflammation process may be particularly effective in these subjects [45].

Metabolic alterations seem key factors in two subgroups in which the alterations are present at a systemic level or with regards only to bone and cartilage metabolism in the affected knee joint [46, 47]. The included studies reporting a metabolic syndrome as key characteristic of a specific KOA subgroup used BMI; blood; and serum biomarkers in their identification process. The use of these features is supported by previous non-phenotyping studies that identified an association between high BMI and OA lesions in non-weight-bearing joints suggesting an underlying systemic pathway [48]. Moreover, recent studies showed that the combination of cardio-metabolic disturbance and obesity increases the risk of OA and identified an association between OA and hypertension, dyslipidaemia, and hyperglycaemia [46, 49–51]. These findings indicate that systemic metabolic alterations could be one of the main causes for the disease in a specific subgroup of subjects. A multi-stages disease model cannot fully explain the existence of a metabolic syndrome subgroup that instead could be explained as a separate KOA phenotype.

Metabolic alterations in the KOA population have been reported not only at a systemic level, but as specific alterations in cartilage and bone metabolism. Biomarker analysis represents the gold standard for the identification of metabolic alterations in bone and cartilage. The identification of specific biomarkers profiles in the KOA population, as emerged from the studies included in this review, which represents strong evidence in support of the existence of a phenotype in which bone and cartilage metabolism are of primary importance as a determinant of the disease. Drugs aiming to influence bone and cartilage metabolism may see their effect improved if tested in this specific phenotype [4].

The possibility of a mechanical overload phenotype emerged from this systematic review; however, a large gap in the evidence regarding the existence of this phenotype emerged, due also to the lack of studies with longitudinal design. Among the studies included, malalignment and muscle strength were the biomechanical variables used to define biomechanical phenotypes [7, 8, 36] in combination with cartilage degeneration, BMI, and previous injuries. Malalignment has been shown to be strongly associated with disease progression and cartilage degeneration in specific compartments of the knee (e.g. varus malalignment is closely associated with medial tibiofemoral compartment disease) [52]; while high muscle strength has been reported as a protective factor against symptomatic but not radiographic KOA [53]. The studies included in this systematic review reported subgroups of KOA subjects with high levels of muscle strength. The authors suggested that the presence of high level of muscle strength in combination with other factors (e.g. malalignment, previous injury, BMI) could signify a group of people with high level of physical activity and biomechanical overload [8]. Therefore, malalignment in combination with other known factors (e.g. muscle strength, previous injury) may confer high local stress in the correspondent joint compartment supporting the hypothesis of biomechanical mechanisms responsible for the disease. For this reason, it is likely that these subjects would respond to, biomechanical interventions (e.g. wedged insoles, knee braces) rather than to drug treatments aiming to protect the cartilage [4].

Although our study aimed to identify phenotypes based on different disease mechanisms, from the literature a group of subjects with low degeneration and mild clinical symptoms emerged. These subjects seem to suggest the existence of a KOA subgroup characterized by minimal joint disease. Although these features could be considered representing an early stage of the disease; three of the included studies showed stability over time (2–10 years) [9, 20, 27], supporting the consideration of this subgroup as a phenotype rather than a stage of the disease. Subjects were classified in this group based on the severity and the outcome of the disease regardless of possible mechanisms or aetiology. Despite this, the clinical characteristics of the subjects classified in this subgroup seem to suggest different underling mechanisms of the disease. The inclusion of outcomes in the classification process makes the identification of subjects belonging to this phenotype difficult in clinical practice. Strong evidence of a clinical variable able to predict the non- progression of the disease is still missing.

In this systematic review, six groups of variables that can indicate the presence of six main phenotypes have been identified. These sets of variables seem to suggest the existence of different disease mechanisms and aetiology in specific subgroups of the KOA population. None of the studies analysed here explored the possibility of an overlap between the suggested subgroups. Considering the variables used to identify phenotypes and the pathophysiology of the disease, there is no reason to exclude the possibility of an overlap. For example, patients with chronic pain could present characteristics considered key factors of other phenotypes like metabolic alterations or malalignment. Therefore, while these phenotypes may be distinct, they are not necessarily mutually exclusive. It can be hypothesized that patients with features consistent with more than one phenotype may be more severely affected by the disease and could be regarded as more complex clinical cases.

Another implication of the overlap between phenotypes is the possibility that the phenotypes identified here do not exist as separate entities in the KOA population, but only as result of the choice of specific variables, samples and analysis in the phenotyping process. This represents a limitation of the review that is not able to conclude if these phenotypes can be regarded as separate entities. Therefore, studies that try to identify KOA phenotypes with different disease mechanisms within the same sample are needed to study the possibility and the entity of overlap between phenotypes and verify the existence of phenotypes as distinct groups. Moreover, studies identifying an overlap between phenotypes may be important in the identification of complex KOA cases that may benefit from a combined treatment approach.

Among the 25 studies included, four had a strong risk of bias [25, 28, 30]. The main source of bias was the presence of confounding factors; of all the studies included in the review, only four studies presented a low risk of bias in that specific area [7, 8, 27, 40]. Disease duration was the main confounding factor taken into account in this systematic review, whereby differences between patients due to them being in different stages of the same disease process could potentially identify subgroups. These disease-stage subgroups did not fit the definition of phenotypes for the purpose of this review. Therefore, studies in which there were significant differences in disease duration between identified subgroups were regarded as at high risk of bias in this area.

Two of the included studies using blood and serum biomarkers in order to identify phenotypes had a mixed sample of KOA and hip OA [24, 37]. In both the samples more than 70 % of the subject had a diagnosis of KOA, but nevertheless findings from these studies should be interpreted with caution when applied to the KOA population.

Another important source of bias was the selection of the study sample. Studies that tried to identify specific phenotype may have oversampled high-risk patients, thus leading to elevated prevalence rates. A similar bias was the inclusion of only patients listed for joint replacement [24, 26, 28]. Furthermore, the evidence presented in this review is limited by the research focus of published studies and their quality. The criterion used to identify a phenotype required the support of two studies with low or moderate risk of bias. This approach implies the possibility that some important phenotypes have not been reported due to a limited number of appropriate studies (as was hypothesized to be the case for the mechanical overload phenotype to some extent).

Because OA is a heterogeneous disease, identifying subgroups for treatments is probably one of the promising ways forward in clinical research [2]. This can only be achieved when the correct methodology to identify such subgroups is used. For this reason, we focused only on studies that had as a main focus the identification of KOA phenotypes. Some studies looking at the influence of specific risk factors of disease progression and outcome were excluded. We are aware that results emerging from these studies may identify useful evidence, especially in generating new hypotheses regarding phenotypes. Nevertheless, the aim of this review was the identification of phenotypes which have already been broadly studied in the literature and that are supported by evidence emerging from these studies. The absence of a post traumatic KOA as an identified phenotype may work as an example. Only Waarsing et al. analysed the rate of knee injuries to characterize their phenotype. Despite the strong evidence that identifies injuries as an important risk factor in the development of KOA; studies investigating whether patients can be meaningfully grouped based on a history of traumatic injury are absent. It may be that subjects with a history of traumatic knee injury constitute a separate phenotype. Alternatively, injuries may predispose patients to KOA through more than one underlying pathway, and may therefore not be a meaningful phenotypic identifier in itself.

The lack of a clear definition of phenotypes makes synthesis of the current literature difficult; therefore, a clear and shared definition of KOA phenotypes would help to better direct future research in the field. To combine studies, we relied on what was reported by the author and on previous research on KOA risk and aetiologic factors. This approach has intrinsic risks and may be affected by a decisional bias. However, all the data used to draw the conclusions have been reported (see Additional file 1) in the attempt to make the decision process as transparent as possible. We found this methodology the best compromise to deal with the large variability in the field and to provide useful evidence. Finally, the six sets of variables identified in this review may not be able to fully explain heterogeneity of the patient population. Future research may yet lead to the identification of different disease mechanisms suggesting the existence of new phenotypes.

## Conclusions

Six main sets of variables suggesting the existence of six clinical phenotypes of KOA characterized by different disease mechanisms were identified in this systematic review: chronic pain; inflammatory; metabolic syndrome; bone and cartilage metabolism; mechanical overload and minimal joint disease. This represents a good starting point for future research aiming to better identify KOA phenotypes. Furthermore, this process of synthesis of evidence may be relevant in the development of better treatment allocation and clinical disease management.

## Additional file

Additional file 1:
**A**. Research strategy. **Table S1**. Key characteristics of subgroups/phenotypes extracted from each study. **Table S2**. Phenotype name reported in the original paper. **Table S3**. Resume of prevalence of the different phenotypes. [7–9, 18–38, 40]. (DOCX 100 kb)

## Abbreviations

BMI: Body mass index
BSP: Bone sialoprotein
COX-2: Cycloxygenase 2
ECP: Eosinophilic cationic protein
ESR: Erythrocyte sedimentation rate
HP: Hydroxylysyl pyridinoline
hsCRP: High-Sensitivity C-reactive protein
IL-1β: Interleukin-1B
IL-8: Interleukin-8
KOA: Knee osteoarthritis
KS: Keratan sulfate
LP: Lysyl pyridinoline
Mets: Metabolic syndrome
MIP-1α: Macrophage inflammatory protein-1α
MIP-1β: Macrophage inflammatory protein-1β
MRI: Magnetic resonance imaging
OA: Osteoarthritis
PIIANP: N-terminal propeptide of collagen IIA
pLeptin: Plasma leptin
pLeptin: Plasma leptin
QST: Quantitative sensory testing
sCOMP: Serum cartilage oligomeric protein
TNFI: Tumor necrosis receptor type I
TNFII: Tumor necrosis receptor type II
