# Supplementary material for: Identification of clinical phenotypes in knee osteoarthritis: a systematic review of the literature
Source: BMC Musculoskelet Disord. 2016 Oct 12;17:425. doi: 10.1186/s12891-016-1286-2 (PMC5062907; doi:10.1186/s12891-016-1286-2)
Supplement: Additional file 1: — A. Research strategy. Table S1. Key characteristics of subgroups/phenotypes extracted from each study. Table S2. Phenotype name reported in the original paper. Table S3. Resume of prevalence of the different phenotypes. [7–9, 18–38, 40]. (DOCX 100 kb) [file 12891_2016_1286_MOESM1_ESM.docx]

Additional file 1

**Supplementary file A**

Research strategy:

("osteoarthritis"[MeSH Terms] OR "osteoarthritis"[All Fields]) AND ("knee"[MeSH Terms] OR "knee"[All Fields] OR "knee joint"[MeSH Terms] OR ("knee"[All Fields] AND "joint"[All Fields]) OR "knee joint"[All Fields]) AND ((phenotyp[All Fields] OR phenotyp3[All Fields] OR phenotypage[All Fields] OR phenotypal[All Fields] OR phenotype[All Fields] OR phenotype'[All Fields] OR phenotype''[All Fields] OR phenotype's[All Fields] OR phenotype1[All Fields] OR phenotypeable[All Fields] OR phenotypeannotation[All Fields] OR phenotyped[All Fields] OR phenotypees[All Fields] OR phenotypegene[All Fields] OR phenotypegenotype[All Fields] OR phenotypein[All Fields] OR phenotypeinvolves[All Fields] OR phenotypeless[All Fields] OR phenotypelevel[All Fields] OR phenotypemarkers[All Fields] OR phenotypeof[All Fields] OR phenotyper[All Fields] OR phenotyperemodeling[All Fields] OR phenotyperepresents[All Fields] OR phenotypers[All Fields] OR phenotypers'[All Fields] OR phenotypes[All Fields] OR phenotypes'[All Fields] OR phenotypes''[All Fields] OR phenotypesin[All Fields] OR phenotypespecific[All Fields] OR phenotypess[All Fields] OR phenotypesthese[All Fields] OR phenotypesto[All Fields] OR phenotypevana[All Fields] OR phenotypexdiet[All Fields] OR phenotypexsurgery[All Fields] OR phenotypextime[All Fields] OR phenotyphic[All Fields] OR phenotyphically[All Fields] OR phenotypia[All Fields] OR phenotypially[All Fields] OR phenotypic[All Fields] OR phenotypic'[All Fields] OR phenotypic2[All Fields] OR phenotypical[All Fields] OR phenotypicality[All Fields] OR phenotypically[All Fields] OR phenotypically'[All Fields] OR phenotypicallyobvious[All Fields] OR phenotypicaly[All Fields] OR phenotypicassays[All Fields] OR phenotypice[All Fields] OR phenotypicically[All Fields] OR phenotypicidentification[All Fields] OR phenotypics[All Fields] OR phenotypictransition[All Fields] OR phenotypie[All Fields] OR phenotypies[All Fields] OR phenotypification[All Fields] OR phenotypificationwas[All Fields] OR phenotyping[All Fields] OR phenotyping'[All Fields] OR phenotypings[All Fields] OR phenotypique[All Fields] OR phenotypiquement[All Fields] OR phenotypiques[All Fields] OR phenotypisation[All Fields] OR phenotypisierung[All Fields] OR phenotypization[All Fields] OR phenotyple[All Fields] OR phenotypoc[All Fields] OR phenotypological[All Fields] OR phenotypology[All Fields] OR phenotypos[All Fields] OR phenotyps[All Fields] OR phenotyptes[All Fields] OR phenotyptic[All Fields] OR phenotypus[All Fields] OR phenotypw[All Fields] OR phenotypy[All Fields] OR phenotypy'[All Fields] OR phenotypying[All Fields]) OR subgroup[All Fields] OR cluster[All Fields] OR "factor analysis"[All Fields])

Table S1: Key characteristics of subgroups/phenotypes extracted from each study.

|  | Phenotypes | | | | | |  |
| --- | --- | --- | --- | --- | --- | --- | --- |
| Author/year | Chronic pain | Inflammatory | Metabolic syndrome | Bone and cartilage metabolism | Mechanical overload | Minimal joint disease | |
| Attur 2011[18] |  | OA DIAGNOSIS: ACR criteria  CHARACTERISTICS: age 60¥, BMI 27. IL-1 gene overexpression of PBLs |  |  |  |  | |
| Bae 2010[19] |  |  |  |  | OA DIAGNOSIS: cadaveric  CHARACTERISTICS: clusters based on cartilage lesion pattern: medial condyle, lateral condyle |  | |
| Berry 2010a[20] |  |  |  | OA DIAGNOSIS: ACR criteria  CHARACTERISTICHS: age 63.7 (10.3)*. 2 groups: high PINP and high osteocalcin in which there was an association between increasing bone resorption markers CTX-I and NTX-I and reduced cartilage loss (2 years) |  |  | |
| Berry 2010b[21] |  |  |  |  |  | OA DIAGNOSIS: ACR criteria  CHARACTERISTICS: age 63.7 (10.3)*. Low COMP and low PIIANP subgroups associated with reduced cartilage loss (2 years) | |
| Blumnenfeld 2013[22] |  |  |  | OA DIAGNOSIS: Chingford cohort CHARACTERISTICS: 55.5 (5.8), BMI 26.4 (4.4); high level of sCOMP and low level of aggrecan predict degeneration (from KL=0 to KL ≥ 2 at 10 years) |  |  | |
| Cruz-Almeida 2013[23] | OA DIAGNOSIS: ACR criteria  CHARACTERISTICS: age Group A: age 56.7 ±6.8 lowest scores on the LOT-R  along with high scores on the PVAQ (low optimism), high level of pain low PPT lower and upper body. Group B: 54.3 ± 5.4 age highest scores  on the PVAQ, KRS, and negative affect subscale of the  PANAS (somatic sensitivity/pain hypervigilance) highest number of pain areas, highest level of pain, lowest PPT lower and upper body. |  |  |  |  |  | |
| Doss 2007[24] |  | OA DIAGNIOSIS: Physician CHARACTERISTICS: Mixed sample: 72% KOA, 28% hip OA .All subjects scheduled for replacement, age 68.7± 7.9*. Elevated IL-6 levels in the synovial fluids |  |  |  |  | |
| Egsgaard 2015[25] | OA DIAGNOSIS: ACR criteria.  CHARACTERISTICS: Low pressure pain  thresholds, enhanced pain responses to temporal summation in all test sites(lower and upper body), high level of pain, high score in pain catastrophizing questionnaire. |  |  |  |  |  | |
| Fernández-Tajes 2014[26] |  | OA DIAGNOSIS: KL 4 and knee replacement.  CHARACTERISTICS:  increased  inflammatory response that regulated by epigenetics |  |  |  |  | |
| Holla 2013[27] |  |  |  |  |  | OA DIAGNOSIS: Check cohort criteria  CHARACTERISTICS: age 56.2±5.2; BMI 25.5±3.6 participants moved from moderate or high levels  of activity limitations at baseline to low levels over 5 years, or  reported permanent low levels of activity limitations. | |
| Jenkins 2015[28] |  |  |  |  |  | OA DIAGNOSIS: Physician  CHARACTERISTICS: age 69 (8.179) undergoing knee replacement, high quality-of-life scores and low WOMAC (indicating higher functional  status) | |
| Kerkhof 2008[29] |  |  |  |  |  |  | |
| Kinds 2013[9] |  |  |  |  |  | OA DIAGNOSIS: Check cohort criteria  CHARACTERISTICS: age 57 (5), BMI 24 (22–27)  No ROA progression (5 years) | |
| King 2013[30] | OA DIAGNOSIS: ACR criteria.  CHARACTERISTICS: age 56.4 (54.8, 58.1) 95% confidence interval; BMI 33.5 (31.9, 35.1) 95% high level of pain, lower PPT and presence of temporal summation (lower and upper body) |  |  |  |  |  | |
| Knoop 2011[7] | OA DIAGNOSIS: OAI progression subcohort.  CHARACTERISTICS: age 63.2 ± 9.1* BMI: 31.0 ± 4.6prevalence of depression in almost all participants (88%), mild to moderate radiographic OA, muscle weakness, and obesity  in 58% of participants |  | OA DIAGNOSIS: OAI progression subcohort.  CHARACTERISTICS: age 63.2 ± 9.1* BMI 35.9 ± 3.5  obesity in all participants (100%), muscle weakness, mild to moderate radiographic OA, low prevalence of depression |  | OA DIAGNOSIS: OAI progression subcohort.  CHARACTERISTICS: age 63.2 ± 9.1* BMI 29.9 ±3.4  High muscle strength, moderate to severe radiographic OA, obesity  in 48% of participants, and low prevalence of depression | OA DIAGNOSIS: OAI progression subcohort  CHARACTERISTICS: age 63.2 ± 9.1* BMI 27.5 ±3.9 mostly no or doubtful evidence of radiographic OA, average muscle strength, low prevalence of obesity and depression | |
| Murphy 2011[31] | OA DIAGNOSIS: reported pain in a joint with OA on the WOMAC scale (Likert version) of ≥ 4 with at least two items rated as moderate pain or higher.  CHARACTERISTICS: age 72.2 (9.8)*; BMI 30.5 (5.9)*; high levels of pain, fatigue, sleep problems, and mood disturbances |  |  |  |  |  | |
| Otterness  2000[32] |  | OA DIAGNOSIS: ACR criteria.  CHARACTERISTICS: age 57*, high level of inflammation biomarkers (IL-6, CRP, TNF RI, TNF RII, and ECP) |  | OA DIAGNOSIS: ACR criteria.  CHARACTERISTICS: age 57*, bone, cartilage and synovial biomarkers divided in 2 factors: 1. epitope 846, HA, CPII; 2. epitope 846, HA, CPII |  |  | |
| Pereira 2013[33] | OA DIAGNIOSIS: KL ≥2. CHARACTERISTICS: Age 58.0 (15.2)* high level of pain, depressive symptoms (BDI ≥14) , no correlation between pain and ROA |  |  |  |  |  | |
| Roemer 2012[34] |  |  |  | OA DIAGNOSIS: MRI (WORMS)  CHARACTERISTICS: hypertrophic phenotype  (large osteophytes with little joint space narrowing) age 67.3 (8.5) BMI 35.9 (4.5), atrophic (no osteophytes with severe joint space loss), age 70.3 (7.9), BMI 28.0 (4.6) |  |  | |
| Sowers 2002[35] |  |  | OA DIAGNOSIS: KL ≥ 2  CHARACTERISTICS: BMI ≥30 and high level of C-RP (people in this group with diabetes had the highest level of C-RP) |  |  |  | |
| Van der Esch 2015 [36] | OA DIAGNOSIS: ACR criteria CHARACTERISTICS: age 61.7 (8.8) BMI 28.5±4.4, muscle strength 28.5 ± 4.4 Nm, Depressive mood. |  | OA DIAGNOSIS: ACR criteria CHARACTERISTICS: age 61.7 (8.8)BMI 41.3 ± 5.0 |  | OA DIAGNOSIS: ACR criteria CHARACTERISTICS: age 61.7 (8.8) BMI 28.8 ± 3.8, Muscle strength 124.3 ± 23.1 Nm | OA DIAGNOSIS: ACR criteria CHARACTERISTICS: age 61.7 (8.8)BMI 27.5±4,0, muscle strength 57.0±20.8 Nm, K/L 0-1 69% | |
| Van Spil 2012[37] |  | OA DIAGNOSIS: CHECK cohort CHARACTERISTICS: mixed sample: 76% KOA, 24% hip OA; age 56 ± 5†, BMI 25.5 (23.3-28.4)† High level of sPIIINP, sHA, sCOMP | OA DIAGNOSIS: CHECK cohort CHARACTERISTICS: age 56 ± 5†, BMI 25.5 (23.3-28.4)†High level of ESR, pLeptin, hsCRP | OA DIAGNOSIS: CHECK cohort CHARACTERISTICS: age 56 ± 5†, BMI 25.5 (23.3-28.4)†High level of uCTX-I, uNTX-I, sPINP, sOC, uCTX-II, |  |  | |
| Waarsing 2015[8] |  |  |  |  | OA DIAGNOSIS: OAI progression subcohort. CHARACTERISTICS1: age 63 lateral compartment, high prevalence of previous injuries (48%) valgus alignment, and low BMI 28.6  2: age 62, high prevalence of previous injuries (55%), severe degeneration medial compartment, BMI 29,4. | OA DIAGNOSIS: OAI CHARACTERISTICS: progression subcohort. Age 60, Low degeneration, medial compartment, neutral alignement, BMI 30,6, low progression (7% for KL^3^ score, 21% for JSN^2^) at 2 years, trauma 33% | |
| Iijima 2015[38] |  |  |  |  | OA DIAGNOSIS: age .50 years,  K/L grade ≥1 in the medial compartment. CHARACTERISTICS: age 74.2 ± 6.42, BMI 25.5, KL1 11.5%, KL2 19.2%, KL 3 38.5%, KL 4 30.8% |  | |
| Kittelson 2015[40] | OA DIAGNOSIS: CHARACTERISTICS: age 64.7, BMI 30.3±5.2, WOMAC score 32.5±20.5, NPRS6.0±2.8, CES-D 14.7±10.6, Number of pain sites 3.1±3.2 |  |  |  |  |  | |

*age of the sample not of the specific group, the author however declared no difference among groups or adjusted the calculation for age.

† : Age was strongly associated with sHA and uCTX-II levels. BMI appeared to be strongly associated with pLeptin levels (SD).

¥: mean age calculated from the data available in the paper.

PBLs: circulating peripheral blood leukocytes.

CSQ-R: Coping Strategies Questionnaire-Revised (CSQ-R). The CSQ-R assesses passive and active coping techniques related to pain (21,22). Participants rated the frequency with which they engage in various coping techniques using a 7-point scale.

LOT-R: Life Orientation Test-Revised (LOT-R). The LOT-R is a 10-item questionnaire assessing dispositional optimism on a 5-point Likert scale.

KRS: Kohn Reactivity Scale (KRS). The KRS is commonly used to measure aspects of hypervigilance and general reactivity and arousability (24) to common experiences across 24 items using a 5-point scale.

PANAS: Positive and Negative Affect Scale (PANAS). The PANAS consists of 20 items rated on a 5-point scale (26,27). High scores on positive affect reflect enthusiasm.

energy, and alertness, while high scores on negative affect reflect distress and aversive mood states.

PVAQ: Pain Vigilance and Awareness Questionnaire (PVAQ). Attention to pain was assessed with the PVAQ (28,29). Consisting of 16 items, participants indicated how frequently they engaged in various behaviors over the past few weeks using a 6-point scale.

STAXI: State-Trait Anger Expression Inventory (STAXI). The STAXI is a 44-item questionnaire designed to evaluate anger (30). Participants rated the frequency/intensity of angry feelings on a 4-point scale. For the present study, the trait subscale was used, reflecting general anger.

CHECK COHORT CRITERIA: Inclusion criteria of the CHECK study are: (1) being aged between 45 and 65 years; (2) pain and/ or stiffness in the knee and/or hip; (3) having at least two of the following clinical criteria for knee OA of the American College of Rheumatology (ACR): knee pain, morning stiffness <30 min, crepitus or bony tenderness ( participants who met this criterion were followed annually); and (4) having had no consultation with a physician for these symptoms (or first consultation was within the 6 months immediately preceding inclusion).

BDI: Beck Depression Inventory.

WORMS: Whole-Organ Magnetic Resonance Imaging Score.

NPRS: numeric pain rating scale.

Table S2: Phenotype name reported in the original paper

|  | Phenotypes | | | | | |  |
| --- | --- | --- | --- | --- | --- | --- | --- |
| Author/year | Chronic pain | Inflammatory | Metabolic syndrome | Bone and cartilage metabolism | Mechanical overload | Minimal joint disease | |
| Attur 2011[18] |  | Group not named |  |  |  |  | |
| Bae 2010[19] |  |  |  |  | 1: MFC*  2:PFG^#^ |  | |
| Berry 2010a[20] |  |  |  | Group not named |  |  | |
| Berry 2010b[21] |  |  |  |  |  | 1: Low COMP  2: Low PIIANP | |
| Blumnenfeld 2013[22] |  |  |  |  |  |  | |
| Cruz-Almeida 2013[23] | Group not named |  |  |  |  |  | |
| Doss 2007[24] |  | Group not named |  |  |  |  | |
| Egsgaard 2015[25] | Pain profile D |  |  |  |  |  | |
| Fernández-Tajes 2014[26] |  | Group not named |  |  |  |  | |
| Holla 2013[27] |  |  |  |  |  | Good outcome group | |
| Jenkins 2015[28] |  |  |  |  |  |  | |
| Kerkhof 2008[29] |  |  |  |  |  |  | |
| Kinds 2013[9] |  |  |  |  |  | No progression group | |
| King 2013[30] | High symptoms group |  |  |  |  |  | |
| Knoop 2011[7] | Depressive phenotype |  | Obese and weak phenotype |  | Strong muscle phenotype | Minimal Joint disease phenotype | |
| Murphy 2011[31] | Group not named |  |  |  |  |  | |
| Otterness  2000[32] |  | Inflammation markers |  | 1: markers of cartilage anabolism  2: markers of cartilage catabolism |  |  | |
| Pereira 2013[33] | Group not named |  |  |  |  |  | |
| Roemer 2012[34] |  |  |  | 1: atrophic phenotype  2: hypertophic phenotype |  |  | |
| Sowers 2002[35] |  |  | Group not named |  |  |  | |
| Van der Esch 2015 [36] | Depressive phenotype |  | Obese phenotype |  | Strong muscle strength phenotype | Minimal Joint disease phenotype | |
| Van Spil 2012[37] |  | Synovium | Inflammation | Bone-CTX-II, |  |  | |
| Waarsing 2015[8] |  |  |  |  | 1: Aggressive OA (medial)  2: Aggressive OA (lateral) | Mild OA | |
| Iijima 2015[38] |  |  |  |  | Malalignment phenotype |  | |
| Kittelson 2015[40] | Psychological distress |  |  |  |  |  | |

*: Medio- femoral cartilage

^#^: Patello-femoral groove

Table S3: resume of prevalence of the different phenotypes.

| Phenotypes | Prevalence |
| --- | --- |
| Minimal joint disease | 26%-47%* |
| Chronic pain | 10%-19% |
| Inflammatory KOA | 22%-30% |
| Metabolic Syndrome | - |
| Bone and Cartilage metabolism | - |
| Mechanical overload | 12%-22% |

*only studies with a longitudinal design has been used to calculate the prevalence. Analysing studies with different design: 17%-47%
